# Surveillance of tick-borne viruses in the border regions of the Tumen River Basin: Co-circulation in ticks and livestock

**DOI:** 10.1371/journal.pntd.0013500

**Published:** 2025-09-04

**Authors:** Zhe Liu, Shengwei Ji, Qiaocheng Chang, Jinqi Wang, Eloiza May Galon, Ying Xu, Guolu Yin, Jixu Li, Xu Gao, Wannian Tian, Zhenzhen Han, Chenghui Li, Zhiqiang Xu, Rui Du, Shujiang Xue

**Affiliations:** 1 Department of Veterinary Medicine, Agricultural College of Yanbian University, Yanji, China; 2 School of Public Health, Shantou University, Shantou, China; 3 College of Veterinary Medicine and Biomedical Sciences, Cavite State University, Cavite, Philippines; 4 Yanbian Center for Disease Control and Prevention, Yanji, China; 5 College of Animal Science, Jilin Agricultural Science and Technology College, Jilin, China; 6 Animal Health and Epidemic Prevention Center, Huludao, China; Oregon State University College of Veterinary Medicine, UNITED STATES OF AMERICA

## Abstract

**Background:**

The unique eco-geographical patterns and climatic conditions of the China-Tumen River border region, combined with frequent cross-border tourism and trade activities, collectively establish this area as a recognized hotspot for tick-borne disease outbreaks. However, critical knowledge gaps persist regarding the eco-epidemiology of emerging tick-borne viruses and the distribution of their potential reservoir hosts within this trinational ecosystem spanning China, North Korea, and Russia.

**Methods:**

We collected a total of 2,004 ticks from the study area, along with blood samples obtained from 42 sheep and 45 cattle. Following viral metagenomic analysis of the ticks, dual verification of target pathogens in all samples was performed using qRT-PCR and RT-PCR assays. Phylogenetic trees were constructed and nucleotide sequences were analyzed to delineate relationships between the obtained virus strains and reference sequences.

**Results:**

Viral metagenomics identified three viruses in ticks: Dabieshan tick virus (DBTV), Songling virus (SGLV), and Yanggou tick virus (YGTV). PCR analysis detected DBTV exclusively in Hunchun ticks (minimum infection rates, MIR:4.73%) and YGTV in Antu specimens (MIR:0.97%). Conversely, SGLV was detected in ticks from all four regions, with MIR of 1.68% (Helong), 0.74% (Hunchun), 1.61% (Antu), and 4.79% (Longjing). Concurrently, SGLV was detected in 19 sheep blood samples from Longjing, yielding a positivity rate of 45.24%, while YGTV was identified in 13 cattle blood samples from Antu, with a positivity rate of 28.89%. Phylogenetically, the DBTV strain clustered with previously reported DBTV and Yongjia tick virus 1 isolates. Sheep-derived SGLV strains shared close evolutionary ties with tick-borne SGLV, whereas YGTV from cattle and ticks formed a distinct cluster with Russian strains but diverged into two branches from Chinese variants, suggesting evolutionary instability.

**Conclusion:**

These findings address critical knowledge gaps in the transmission dynamics and genetic diversity of emerging arboviruses while providing vital insights for developing cross-border surveillance strategies with significant public health implications.

## Author summary

The ecologically complex Tumen River Basin, a tri-national ecoregion at high risk for tick-borne disease emergence, remains poorly characterized in terms of enzootic arbovirus transmission dynamics. To address this gap, we conducted integrated surveillance of ticks and livestock across four border counties in China. Viral metagenomics revealed three emerging tick-borne viruses (DBTV, SGLV, and YGTV) in tick populations, with PCR validation confirming their distinct spatial distributions. Notably, SGLV exhibited potential for cross-species transmission, as evidenced by its detection in Longjing sheep populations, while YGTV demonstrated host-specific tropism toward cattle.This study provides the first evidence of YGTV and SGLV spillover into domestic animals in Northeast Asia and highlights the Tumen Basin as a hotspot for arbovirus evolution. Our findings underscore the urgent need for coordinated, transnational One Health surveillance across China-North Korea-Russia border zones to mitigate emerging zoonotic threats.

## 1. Introduction

Ticks are obligate hematophagous arthropods that transmit diverse pathogens, including bacteria, viruses, and protozoa [1–3]. Globally, over 900 tick species are classified into three families: Ixodidae, Argasidae, and Nuttalliellidae [4],with 124 species identified in China. Alongside the increasing frequency of international trade, ongoing climate change, and anthropogenic activities, the incidence of tick-borne viruses (TBVs) has increased worldwide, posing a significant threat to public health and livestock development. With growing attention to TBVs and advancements in viral metagenomic sequencing technologies, an increasing number of novel TBVs have been discovered, including Alongshan virus (ALSV), Songling virus (SGLV), Yanggou virus (YGTV), Dabieshan virus (DBTV), and Beiji virus (BJNV) [5–10].

*Jingmenvirus* group (JMV) was first isolated from *Rhipicephalus microplus* [11]. The virus has now been detected across multiple provinces in China, with evidence of human infection [12]. Furthermore, JMV has been identified in diverse tick species, mosquito vectors, rodents, and other mammalian hosts [5,13,14]. In recent years, studies from various countries have reported JMV in different tick species, highlighting its potential threat to public health [7,15–17]. ALSV and YGTV belong to the JMV within the *Flaviviridae* family, with their genomes consisting of four segments, among which segments S1 and S3 exhibit homology to the NS3 and NS5 protein-coding regions of non-segmented flaviviruses. The segments S2 and S4 are uniquely specific to JMVs [18]. Initially identified in *Dermacentor* ticks from China, YGTV has since been reported in D*ermacentor marginatus* and *Dermacentor nuttalli* from two adjacent regions in Central Asian Russia [6], and *I. persulcatus* along the China-North Korea border [19]. However, information regarding other potential hosts of YGTV remains scarce.

SGLV, belonging to the *Orthonairovirus* genus of the *Nairoviridae* family. Its genome comprises three single-stranded negative-sense RNA segments (S, M, and L), which encode the nucleoprotein (NP), glycoprotein precursor, and RNA-dependent RNA polymerase (RdRp), respectively. Owing to its high conservation and abundant expression among viruses, NP is frequently utilized as a target for molecular detection [20].This virus was first identified in 2021 from a patient in Heilongjiang Province, China, who had been bitten by a tick [21]. Recent surveillance has now confirmed its presence in *H. concinna* collected from areas along the China-North Korea border [8]. SGLV infection has been confirmed to induce symptoms such as fever and headache [22], highlighting the existence of numerous TBVs that remain uncharacterized but are capable of infecting humans. These viruses may pose a significant potential threat to both human and animal health. Moreover, SGLV infection in animals has been detected in *Rhombomys opimus* from Xinjiang Uygur Autonomous Region in northwestern China [23].

DBTV was first identified in 2015 in *Haemaphysalis longicornis* ticks in Hubei Province, China [24], and subsequently reported for the first time in Japan in 2023 [25]. The RNA genome of the virus is composed of S and L segments, distinguishing it from typical phlebovirus by the absence of the M segment [26]. Current studies have confirmed that DBTV can be transmitted transovarially and transstadially in *H. longicornis*, which is known to parasitize various bird species, including migratory birds [10]. The virus is widely distributed across regions such as South Korea, Japan, and multiple provinces in China, including Shandong and Hubei [25,27–30].

The Tumen River system, one of China’s significant international rivers, borders the northern part of the Korean Peninsula and the Far East region of Russia, encompassing areas of China, Russia, and North Korea. In this pivotal region, the three nations-China, Russia, and North Korea-have engaged in extensive international regional cooperation. Consequently, investigating the prevalence and distribution of TBVs in these border areas is of paramount importance [31]. The basin is characterized by a temperate continental monsoon climate, which supports a rich diversity of flora and fauna [32]. Transboundary migratory wildlife and intensively grazed livestock serve as mobile vectors for cross-species pathogen transmission, thereby facilitating favorable conditions for tick proliferation and TBVs dissemination [19,32–36]. Concurrently, occupational exposure among farmers/herders and expanding tourism activities further amplify the probability of human-tick contact. It is crucial to elucidate the composition of TBVs in this region. This study employed viral metagenomic to systematically characterize the virome of ticks collected from China’s border region along the Tumen River basin, with parallel surveillance of viral pathogens in domesticated ruminants (cattle and sheep) within the same ecological zone. This research aims to address critical knowledge gaps regarding TBVs in this region, providing a scientific foundation for the precise prevention and control of tick-borne diseases in border areas, thereby effectively mitigating the risk of cross-border disease transmission.

## 2. Materials and methods

### 2.1 Ethics statement

All animal experiments in this study were approved by the Laboratory Animal Ethics Committee Yanbian University, the Institutional Animal Care and Use Committee (IACUC) Issue No. is YD20250514002.

### 2.2 Tick and blood sample collection

**Fig 1:**
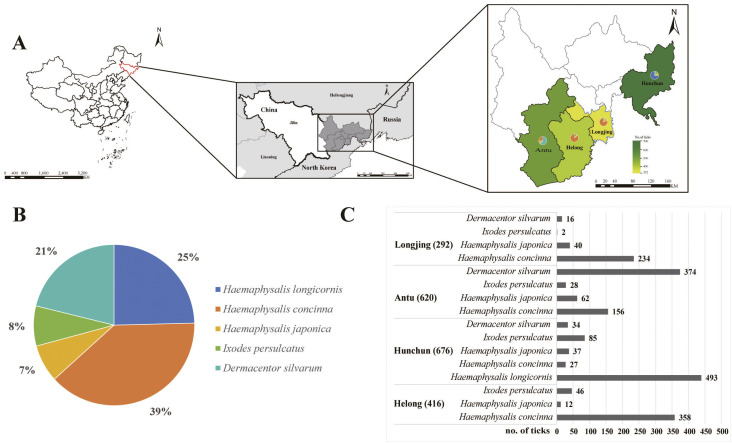
Overview of tick sample collection. Location of Antu, Helong, Longjing, Hunchun where samples were collected **(A)**. The proportion (B) and number (C) of different tick species used in the study are presented in the pie and bar graphs, respectively. The map was constructed using ArcGIS v10.8.2 software. The basemap shapefiles were downloaded from the Chinese Resource and Environmental Science Data Platform (http://www.resdc.cn/).

From April to July 2023 and 2024, ticks were collected from four regions within the Tumen River Basin using the woolen flannel cloth dragging method (Fig 1A). The morphological features of ticks were examined under a stereomicroscope morphologically [4], followed by confirmation through sequencing of the mitochondrial cytochrome c oxidase subunit I (COI) gene [37]. A 97% concordance rate (97/100) was observed between morphological and COI gene sequencing-based identifications of tick specimens. All the ticks were divided into 278 pools based on their collection sites, species, and sex, with each pool consisting of 10 ticks, then stored at −80°C for subsequent analysis. With the assistance of professionals from the local Center for Disease Control and Prevention (CDCP), blood samples from sheep in Longjing City and cattle in Antu City were collected using the jugular vein blood sampling method. The collected blood samples were stored at 4°C overnight.

### 2.3 Processing of tick and blood samples

The tick samples were washed using 1% commercial bleach and sterilized ddH_2_O [38]. For each pool, 400 μL of Dulbecco’s Modified Eagle’s Minimum Essential Medium (DMEM) and four zirconium beads(3 mm diameter) were added, and crushed using the high-throughput tissue grinder (Jingxin, Shanghai, China) for 10 minutes at a frequency of 60 Hz. The homogenate was then centrifuged at 13,400 × g for 10 min at 4°C. The supernatant was aspirated and filtered using 0.45 μm and 0.22 μm Millex filters (Millipore, USA). Approximately 150 μL aliquots of the clarified supernatant from each pool were transferred into newly labeled 1.5 mL microcentrifuge tubes, and viral RNA was extracted using the QIAamp Viral RNA Mini Kit (Qiagen, Germany), according to the manufacturer’s instructions. Subsequently, the quality and concentration of the extracted RNA were assessed using an SMA 1000 UV spectrophotometer (Merinton, China).

For the blood samples, the clotted blood was centrifuged at 5,180 × g for 5 min at 4°C to prepare the serum. Total RNA was extracted using the TIANamp Virus RNA Kit (TIANGEN BIOTECH Co., Ltd.) and stored at −80°C.

### 2.4 Library construction and sequencing

The supernatants from the 278 pools were divided into four groups based on their regional origins. First strand viral cDNA was synthesized using SuperScript Reverse Transcriptase (Thermo, China) and 100 pmol/μL of anchored random primers. Subsequently, double-stranded cDNA was generated from the sscDNA using the Klenow Fragment (TaKaRa, China). Single-primer PCR amplification was then performed using barcoded random primers (S1 Table). The PCR products from each sample were then purified using the QIAquick PCR Purification Kit (Qiagen, Germany) and eluted in 50 μL of TE buffer (100 mM Tris-HCl, 10 mM EDTA, pH 8.0) [39].

### 2.5 Bioinformatics analysis

The four groups of labeled and purified PCR products were sent to Shanghai Personalbio Technology Co., Ltd., where viral metagenomic sequencing was performed using the Illumina NovaSeq 6000 high-throughput sequencing platform. The raw sequencing data were saved in FASTQ format. The raw data were filtered and quality-controlled using fastp (v0.20.0). Subsequently, the quality-controlled valid sequences were aligned to the host sequences using the minimap2 software to remove host-derived contaminating sequences [40]. The remaining reads were assembled using MEGAHIT [41], with contigs of at least 300 bp in length retained by default. Subsequently, the assembled reads and contigs were compared against the NCBI nr and refseq-viral databases using Kaiju [42] and diamond blastp, with an e-value threshold set at 1 × 10^-5^ [43,44]. The highest BLAST hit value within each group was selected [28]. To amplify the full-length sequence of the virus, primers were designed using Primer 6 (Premier Biosoft International, USA). RT-PCR was employed to bridge the sequence gaps between contigs, and the resulting PCR products were subsequently sequenced using the Sanger sequencing method [45].

Phylogenetic trees were reconstructed using the neighbor-joining method in MEGA11 software [46]. Nucleotide sequences of viral isolates from this study were aligned with representative reference sequences accessed from GenBank. Bootstrap values greater than 70, based on 1,000 bootstrap replicates, were displayed on the constructed tree.

### 2.6 PCR detection of pathogens in samples

To validate the results of metagenomic sequencing and determine the prevalence of DBTV, YGTV, and SGLV in the samples, the presence of these viruses was confirmed using qRT-PCR and RT-PCR, respectively. All primers and probes targeting DBTV, SGLV, and YGTV utilized in this study are provided in S2 Table. All qRT-PCR-positive samples were further validated using RT-PCR. Positive samples were identified by visualization with SYBR Safe (Thermo, Waltham, USA) after 1% agarose gel electrophoresis, followed by sequencing for confirmation.

### 2.7 Statistical analysis

Statistical analyses were conducted using IBM SPSS Statistics 26.0. Categorical variables were expressed as absolute frequencies with percentages. Chi-square tests were employed to compare the prevalence rates of three target pathogens among distinct geographical regions and tick species, with statistical significance defined as *P* < 0.05.

## 3. Results

### 3.1 Sample characteristics

A total of 2,004 ticks, including 493 *Haemaphysalis longicornis*, 775 *Haemaphysalis concinna*, 151 *Haemaphysalis japonica*, 161 *Ixodes persulcatus*, and 424 *Dermacentor silvarum*, were collected from the Tumen River Basin. *H. concinna* was the dominant tick species. Geographically, 620 ticks were collected from Antu County, 676 from Hunchun City, 416 from Helong City, and 292 from Longjing City (Fig 1B and 1C). The ticks were divided into 278 pools for subsequent analysis (S3 Table). In the Tumen River Basin, 42 sheep blood samples were collected from Longjing City, and 45 cattle blood samples were obtained from Antu County.

### 3.2 Taxonomic classification

According to the sampling sites, the homogenates were grouped into four mixtures for NGS analysis, each consisting of 40–107 pools. In total, 90 Gb of raw nucleotide data were obtained, of which 96.3% to 96.64% were high-quality (>Q20). Each library generated approximately 144,968,692–1,615,502,212 clean reads, from which 82,338–1,154,586 viral reads were identified, accounting for approximately 0.056% to 0.071% of the total reads. Following de novo assembly, a total of 4,526 viral contigs were obtained. The analysis using Kaiju and Diamond blastp revealed the existence of three viruses, namely DBTV, SGLV, and YGTV. All nucleotide sequences obtained in this study have been submitted to the NCBI database with the GenBank accession numbers: PV034568- PV034579.

### 3.3 Viral detection rates

DBTV was exclusively detected in *Haemaphysalis longicornis*, while YGTV demonstrated restriction to *Dermacentor silvarum*. In contrast, SGLV exhibited broader tropism, being identified across three ixodid species - *Haemaphysalis longicornis*, *Haemaphysalis concinna*, and *Ixodes persulcatus*. Based on 32, 6, and 36 positive pools out of 278 pools derived from 2,004 ticks, DBTV, YGTV, and SGLV were detected with minimum infection rates (MIRs) of 1.59% (32/2,004), 0.30% (6/2,004), and 1.80% (36/2,004), respectively. Analyzed by tick species, the MIR of DBTV in *H. longicornis* was 6.49% (32/493), with a viral RNA concentration of approximately 2.67 × 10^5^ copies/μL. YGTV exhibited an MIR of 1.42% (6/424) in *D. silvarum.* For SGLV, the MIRs were 1.42% (7/493) in *H. longicornis*, 3.35% (26/775) in *H. concinna*, and 1.86% (3/161) in *I. persulcatus*, with a viral RNA concentration of approximately 6.479 × 10^5^ copies/μL (Table 1). Geographically, DBTV was detected solely in tick samples from Hunchun, with an MIR of 4.73% (32/676). YGTV was found only in tick samples from Antu, with an MIR of 0.97% (6/620). SGLV exhibited MIRs of 1.68% (7/416) in Helong, 0.74% (5/676) in Hunchun, 1.61% (10/620) in Antu, and 4.79% (14/292) in Longjing. Despite the close geographical proximity of these locations, the prevalence rates of DBTV, YGTV, and SGLV varied significantly (Table 2) (S4 Table).

**Table 1:** Regional variations in prevalence rates of three Tick-borne viruses across five tick species of the Tumen River Basin.

Viruses	Viruses positivity rate					Value	*P*
	H. longicornis	*H. concinna*	*I. persulcatus*	*D. silvarum*	H. japonica		
Dabieshan tick virus	6.49% (32/493)	0	0	0	0	X^2^ = 99.669	< 0.001
Songling virus	1.42% (7/493)	3.35% (26/775)	1.86% (3/161)	0	0	X^2^ = 21.588	< 0.001
Yanggou tick virus	0	0	0	1.42% (6/424)	0	X^2^ = 22.426	< 0.001

**Table 2:** Regional variations in prevalence rates of three Tick-borne viruses across four areas of the Tumen River Basin.

Viruses	Viruses positivity rate				Value	*P*
	Hunchun	Helong	Longjing	Antu		
Dabieshan tick virus	4.73% (32/676)	0	0	0	X^2^ = 63.884	< 0.001
Songling virus	0.74% (5/676)	1.68% (7/416)	4.79% (14/292)	1.61% (10/620)	X^2^ = 19.306	< 0.001
Yanggou tick virus	0	0	0	0.97% (6/620)	X^2^ = 13.434	0.004

Based on the results of qRT-PCR analysis, the present study identified 19 out of 42 sheep serum samples collected from Longjing as positive for SGLV, representing a positivity rate of 45.24%, with a viral RNA concentration of approximately 1.453 × 10^4^ copies/μL. Additionally, among 45 cattle serum samples collected from Antu, 13 were found to be positive for YGTV by RT-PCR, yielding a positivity rate of 28.89%. Our findings revealed the presence of YGTV in both tick and livestock samples collected from the Antu region. Similarly, SGLV was detected in samples obtained from the Longjing region, suggesting that SGLV may also be widely distributed among livestock in the Hunchun, Antu, and Helong regions.

### 3.4 Phylogenetic analysis

**Fig 2:**
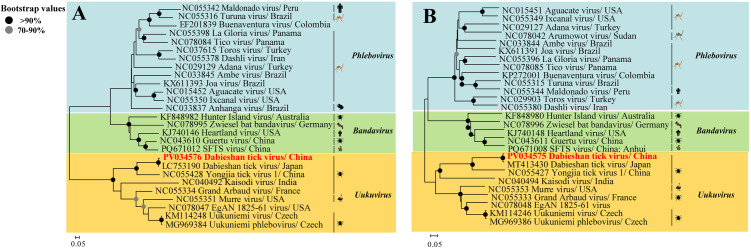
Phylogenetic analyses of the S and L segments of the Dabieshan tick virus. The analyses involved an almost complete nucleotide sequence of the S (1,781 bp) (A) and L (6,520 bp) (B) segments of the DBTV. GenBank accession numbers are listed for each strain/amplicon. The DBTV sequences determined in this study were submitted to the NCBI GenBank under accession nos. PV034575 and PV034576. All viral strains obtained in this study were highlighted in red and bold to distinguish them from the reference sequences (Created with https://commons.wikimedia.org).

The complete coding genome of DBTV was sequenced from *H. longicornis* collected in Hunchun, Yanbian. The S and L segments were identified to be 1,751 bp and 6,520 bp in length (designated JLYB-2024–1/2 tick virus), respectively. Nucleotide sequence alignment analysis with other reference strains revealed that the S segment sequence (PV034576) exhibited the highest nucleotide identity of 99.1% with the DBTV strain from *H. longicornis* in Japan (LC753190) (S5 Table). In contrast, the nucleotide identity with Yongjia tick virus 1 (NC055428), Uukuniemi virus (KM114248), and Kaisodi virus (NC040492) was 59.0%, 41.7%, and 40.9%, respectively. The 6,520 bp L segment sequence (PV034575) demonstrated 98.8% and 65.5% nucleotide identity with DBTV (MT413430) and Yongjia tick virus 1 (NC055427), respectively (S6 Table), and was phylogenetically closest to the Shandong strain (MT413430) (Fig 2B). Based on the S/L segment of JLYB-2024–1/2 tick virus, a neighbor-joining phylogenetic tree was constructed. The analysis revealed that the Yanbian strain of DBTV clustered closely with DBTV and Yongjia tick virus 1, exhibiting the closest phylogenetic relationship with DBTV, while showing a more distant relationship with other viral strains within the same cluster (Fig 2A and 2B).

**Fig 3:**
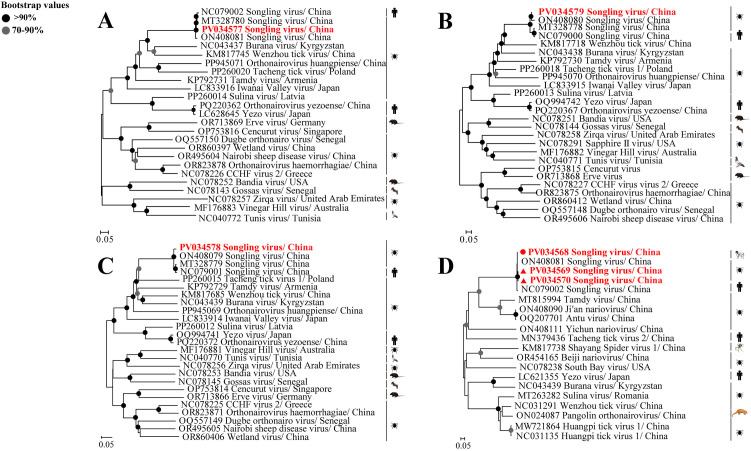
Phylogenetic analysis of the identified Songling tick virus. The analyses involved an almost complete nucleotide sequence of the S (1,885 bp) **(A)** M (4,335 bp) (B) and L (12,002 bp) (C) segments of the SGLV. Phylogenetic analysis of the sequences of SGLV S segment (500 bp) amplified from qRT-PCR positive tick and sheep samples **(D)**. The SGLV sequences determined in this study were submitted to the NCBI GenBank under accession nos. PV034577, PV034579, PV034578, PV034568, PV034569, and PV034570. All viral strains obtained in this study were highlighted in red and bold to distinguish them from the reference sequences. Red circles indicate the virus strains obtained from sheep, while red triangles indicate the virus strains obtained from ticks described in this study (Created with https://commons.wikimedia.org).

The S, M, and L segments of the Yanbian strain of SGLV obtained from tick samples were determined to be 1,885 bp (PV034577), 4,335 bp (PV034579), and 12,002 bp (PV034578) in length, respectively, and were designated as JLYB-2024–3/4/5 tick virus. Nucleotide sequence alignment analysis revealed that the Yanbian strain of SGLV (PV034577) exhibited 97.5% and 97.3% nucleotide identity in the S segment with the SGLV Heilongjiang Lanxi strain (NC079002) and the SGLV Heilongjiang Tahe strain (ON408081), respectively (S7 Table). In comparison, the highest nucleotide identity of 98.4% was observed with the Heilongjiang Yichun (MT328780). The M segment of the Yanbian strain of SGLV (PV034579) exhibited the highest nucleotide identity of 94.9% with the SGLV Heilongjiang Tahe strain (ON408080) (S8 Table), while for the L segment, the Yanbian strain of SGLV (PV034578) showed 96.3% and 96.8% nucleotide identity with the SGLV Heilongjiang Lanxi strain (NC079001) and the Heilongjiang Yichun strain (MT328779), respectively, with the highest nucleotide identity of 97.0% observed with the SGLV Heilongjiang Tahe strain (ON408079) (S9 Table). Phylogenetic analysis revealed that the Yanbian strain of SGLV clustered within the same evolutionary branch as previously reported SGLV strains (Fig 3A-3C). This indicates a close genetic relationship between the strain obtained in this study and those identified in Heilongjiang, while demonstrating a more distant relationship with other orthonairoviruses within the same evolutionary group. Furthermore, phylogenetic trees constructed from the S (Fig 3A), M (Fig 3B), and L (Fig 3C) segments of the SGLV Yanbian strain exhibited nearly identical branching patterns. Analysis of the S segment revealed that the Yanbian strain clustered with the Heilongjiang Yichun strain, while both the M and L segments of the Yanbian strain showed closest phylogenetic relationships with the Heilongjiang Tahe strain (Fig 3A-3C).

Furthermore, three qRT-PCR-positive samples were analyzed for amplification and sequencing of the partial S segment of SGLV (S2 Table). The results demonstrated that SGLV strain derived from sheep (PV034568) and ticks (PV034569, PV034570) exhibited 96.4%-99.6% nucleotide identity in the partial S segment (500 bp), indicating a close phylogenetic relationship between these isolates. Among them, the SGLV strain from sheep (PV034568) showed the highest nucleotide identity of 99.0% with the Heilongjiang *H. concinna* strain (ON408081), followed by the Yanbian SGLV strain (PV034569) with 98.7% nucleotide identity (S10 Table), indicating high sequence similarity among SGLV strains from different provinces and suggesting relative evolutionary conservation of SGLV The phylogenetic analysis revealed that the viral strains obtained in this study formed a monophyletic cluster with SGLV reference strains from GenBank (Fig 3D), sharing a nucleotide sequence identity of 95.5%-99.6% (S10 Table).

**Fig 4:**
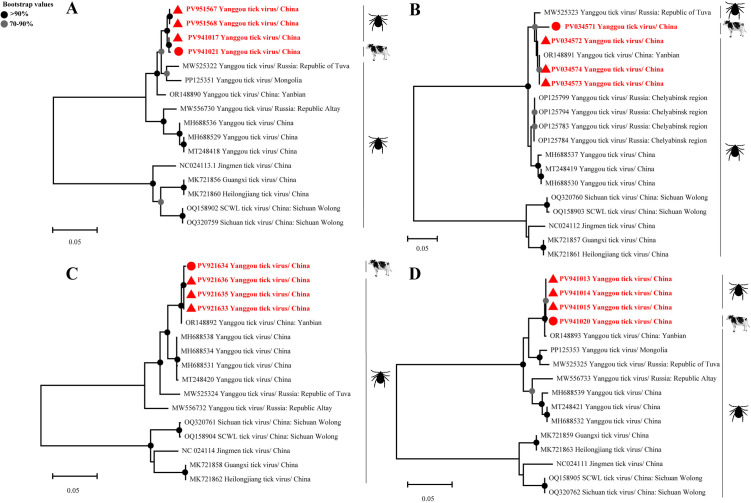
Phylogenetic analysis of the identified Yanggou tick virus. The analyses targeted partial sequences of four YGTV segments: segment 1 (683 bp) **(A)**, segment 2 (305 bp) **(B)**, segment 3 (1,130 bp) **(C)**, and the segment 4 (379 bp) **(D)**. The YGTV sequences determined in this study were submitted to the NCBI GenBank under accession nos. PV951567, PV951568, PV941017, PV941021, PV034571-74, PV921633-36, PV941013-15, PV941020. All viral strains obtained in this study were highlighted in red and bold to distinguish them from the reference sequences. Red circles indicate the virus strains obtained from cattle, while red triangles indicate the virus strains obtained from ticks described in this study (Created with https://commons.wikimedia.org).

Furthermore, we successfully amplified and sequenced four partial YGTV genomic segments from RT-PCR-positive samples (Segment 1: PV951567, PV951568, PV941017, PV941021 (683 bp); Segment 2: PV034571-PV034574 (305 bp); Segment 3: PV921633-PV921636 (1130 bp); Segment 4: PV941013-PV941015, PV941020 (379 bp)). Nucleotide sequence alignment demonstrated that Segments 1–4, amplified from both ticks (PV951567, PV951568, PV941017, PV034572-PV034574, PV921633, PV921635-PV921636, PV941013-PV941015) and cattle (PV941021, PV034571, PV921634, PV941020), exhibited the highest homology with the corresponding regions of the YGTV Yanbian reference strain (OR148890-OR148893), indicating a highly conserved prevalent strain circulating widely in the Yanbian region. Notably, sequences derived from cattle and ticks showed exceptionally high pairwise homology (95.4-100.0%), supporting sustained viral co-circulation within a tick-cattle transmission cycle. Although all segments displayed maximal homology with the Chinese Yanbian strain, distinct secondary homology relationships were identified: Segment 1 and Segment 2 showed the next highest homology with the Russian strain, Segment 3 with a Chinese strain, and Segment 4 with a Mongolian strain (S11–S14 Tables). Phylogenetic analysis based on the sequences of Segments 1–4 demonstrated that our amplified YGTV strains clustered tightly with the Chinese Yanbian strain, forming a distinct, highly supported monophyletic clade (Fig 4). Within this clade, cattle- and tick-derived sequences were interspersed, suggesting minimal evolutionary divergence during cross-species transmission, necessitating expanded serological monitoring in additional livestock hosts, including horses and swine, to characterize the scope of viral host tropism. Critically, this clade also contained strains from Russia and Mongolia, confirming the potential for transboundary spread and underscoring the need for ongoing surveillance of cross-host and cross-border transmission dynamics.

## Discussion

The Tumen River originates from Tianchi Lake (Heavenly Lake) on Paektu Mountain (Changbai Mountain) in Jilin Province, China, traversing China, North Korea, and Russia before discharging into the Sea of Japan. This unique geographical position establishes it as a critical ecological corridor within the tri-border region [31]. Our tick- and livestock-associated epidemiological survey in border areas of the Tumen River basin revealed the detection of DBTV, YGTV, and SGLV in tick specimens. Notably, YGTV and SGLV were first identified in cattle and sheep serum samples, respectively. These findings indicate substantial transmission risks of TBVs in this interconnected ecosystem, necessitating enhanced surveillance of arthropod vectors and zoonotic interfaces.

This study presents the first detection of DBTV in *H. longicornis* ticks collected from the China-Russia-North Korea border region, specifically in Hunchun, Jilin Province, confirming their role as natural reservoirs for this pathogen [25]. Although previous studies in China have reported DBTV genomic sequences in *Rhipicephalus* spp. [47], notably, no *Rhipicephalus* specimens were collected during our investigation. Phylogenetic analysis revealed high shared identity in the S segment of the Yanbian DBTV strain and the 2023 Japanese strain. This high nucleotide identity suggests potential transboundary transmission of TBVs between these regions, likely facilitated by environmental and climatic changes coupled with wildlife migration patterns [48]. Our findings highlight the importance of continuous surveillance at geographical convergence zones to monitor emerging arboviral threats. Notably, migratory birds, serving as parasitic hosts for *H. longicornis* [49], have been implicated in the transcontinental spread of TBVs. Previous studies have documented that the overseas dispersal routes of severe fever with thrombocytopenia syndrome virus (SFTSV) from South Korea to Japan and China’s Zhejiang Province align with migratory bird flyways [50], further corroborating their role in facilitating the long-range dissemination of tick-borne pathogens. Strikingly, among the four sampling sites, DBTV positivity was exclusively detected in Hunchun, whereas all three other locations yielded negative results. This variation in DBTV prevalence across distinct sampling sites may suggest a correlation between tick-borne viral transmission and ecological and geographical determinants [51]. Hunchun might harbor unique ecological drivers-such as climate, temperature, and humidity-that support sustained viral circulation. Moreover, an alternative explanation for our findings could be the absence of *H. longicornis* specimens collected in Helong, Longjing, and Antu regions during sampling. This primarily resulted from *H. longicornis* being neither the dominant tick species nor abundant in these areas, whereas Hunchun’s ecological conditions favor its survival and reproduction. Future studies will prioritize sampling *H. longicornis* in these three regions to analyze the relative contributions of vector species and geographic factors to viral distribution. Among 107 tick pools comprising 676 individual ticks collected in Hunchun, 32 pools tested positive for DBTV, yielding a MIR of 4.73%. Previous studies have detected DBTV in sheep sera from Shandong Province suggests that livestock may act as amplifying hosts [52]. The higher DBTV prevalence observed in Hunchun may indicate the establishment of stable tick-borne virus transmission cycles in this area, representing a significant public health concern that warrants immediate attention.

SGLV was detected in tick specimens from all four sampling sites, suggesting its widespread prevalence in the Tumen River basin. Notably, significant differences in SGLV positivity rates were observed among the four locations, with Longjing District showing a relatively higher infection rate of 4.79% compared to other regions. Interestingly, phylogenetic analysis revealed that the S, M, and L segments of SGLV identified in ticks exhibited distinct genetic divergence from human-derived viral sequences reported in Heilongjiang Province, China, indicating substantial geographic heterogeneity in viral genetic diversity [21]. This observation aligns with the evolutionary characteristics of viruses, which typically co-evolve with host organisms over extended evolutionary timelines while maintaining adaptive potential for cross-species transmission [24,53]. Given the high positivity rate detected in tick specimens from Longjing, we subsequently collected and analyzed sheep blood samples from the same region. The molecular testing revealed 19 SGLV-positive cases among 42 sheep serum samples (45.24%). As SGLV has been classified as a zoonotic pathogen [21], the remarkably high seroprevalence observed in domestic livestock necessitates expanded multispecies surveillance across different geographical regions to better understand its epidemiological patterns.

As a member of the *Jingmenvirus* group within the family *Flaviviridae*, YGTV has been previously isolated from engorged ticks collected from cattle, with arthropods typically acquiring infection through feeding on vertebrate hosts during the viremic phase [54]. Nevertheless, emerging evidence suggests that tick-borne flaviviruses are capable of non-viremic transmission, bypassing the classical viremia-dependent pathway. [55]. Collectively, these findings demonstrate the intricate and multifaceted host-pathogen interactions characteristic of flaviviruses [56–58]. Emerging evidence has established the human pathogenicity of JMTV and ALSV, as documented in clinical reports [12,59]. In contrast, the pathogenicity of YGTV in both animals and humans remains undetermined. Our surveillance detected YGTV in both tick specimens and cattle serum samples from Antu County, while no positive cases were identified in three adjacent regions. Intriguingly, The amplified YGTV fragments demonstrated significant heterogeneity in their phylogenetic relationships. Consistent with its segmented RNA genome, YGTV permits independent evolution of individual genomic segments. This segment-specific divergence pattern provides robust evidence for a complex network of tick-borne viral migration and recombination circulating within transboundary zones of the Tumen River Basin. These findings underscore the necessity to evaluate the virus’s potential zoonotic risk through enhanced host surveillance coupled with molecular phylogeny-based tracing. Of particular note, although YGTV remains understudied, *Dermacentor* tick species, recognized as its primary vectors, exhibits a broad geographical distribution across Asia and Europe, suggesting potential dissemination of this virus beyond currently identified endemic zones. These findings underscore the need for enhanced surveillance of YGTV’s adaptive mutations and cross-species transmission risks [6,60,61].

## Conclusions

This study documents the detection of DBTV, SGLV, and YGTV in tick populations across the Tumen River Basin, providing key epidemiological data on their distribution. Significantly, SGLV and YGTV were respectively identified in sheep and cattle specimens from this region, marking the first evidence of these viruses in domestic ruminants within this region. These findings advance our understanding of TBVs composition in border ecosystems and underscore the need to delineate reservoir host spectra and natural transmission cycles for these emerging arboviral pathogens.

## Supporting information

S1 Table Primers for macrogenomic analysis. (DOCX)

S2 Table Primers and probes used in the present study. (DOCX)

S3 Table Tick species for DBTV, SGLV, and YGTV collected in Tumen River basin area, China. (DOCX)

S4 Table Positive rates of tick samples for DBTV, SGLV and YGTV collected in Tumen River basin area,China. (DOCX)

S5 Table Pairwise comparison (%) of nucleotide identity for the S segment of Dabieshan tick virus in the study. (DOCX)

S6 Table Pairwise comparison (%) of nucleotide identity for the L segment of Dabieshan tick virus in the study. (DOCX)

S7 Table Pairwise comparison (%) of nucleotide identity for the S segment of Songling tick virus in the study. (DOCX)

S8 Table Pairwise comparison (%) of nucleotide identity for the M segment of Songling tick virus in the study. (DOCX)

S9 Table Pairwise comparison (%) of nucleotide identity for the L segment of Songling tick virus in the study. (DOCX)

S10 Table Pairwise comparison (%) of nucleotide identity for the protein S segment of Songling tick virus in the study. (DOCX)

S11 Table Pairwise comparison (%) of nucleotide identity for the protein 1 segment of Yanggou tick virus in the study. (DOCX)

S12 Table Pairwise comparison (%) of nucleotide identity for the protein 2 segment of Yanggou tick virus in the study. (DOCX)

S13 Table Pairwise comparison (%) of nucleotide identity for the protein 3 segment of Yanggou tick virus in the study. (DOCX)

S14 Table Pairwise comparison (%) of nucleotide identity for the protein 4 segment of Yanggou tick virus in the study. (DOCX)
