# Supplementary material for: Surveillance of tick-borne viruses in the border regions of the Tumen River Basin: Co-circulation in ticks and livestock
Source: PLoS Negl Trop Dis. 2025 Sep 4;19(9):e0013500. doi: 10.1371/journal.pntd.0013500 (PMC12419658; doi:10.1371/journal.pntd.0013500)
Supplement: S1 Table — (DOCX) [file pntd.0013500.s001.docx]

**S1 Table. Primers for macrogenomic analysis**

| Primers type | Primers number | Number (5'-3') |
| --- | --- | --- |
| Anchored Random Preimers | RT1 | GCCGGAGCTCTGCAGATATCNNNNNN |
|  | RT2 | GTATCGCTGGACACTGGACCNNNNNN |
|  | RT3 | CATCACATAGGCGTCCGCTGNNNNNN |
|  | RT4 | CGCAGGACCTCTGATACAGGNNNNNN |
|  | RT5 | CCGAGGTTCAAGCGAGGTTGNNNNNN |
|  | RT6 | GGTGGGCGTGTGAAATCGACNNNNNN |
|  | RT7 | CGACCCTCTTATCGTGACGGNNNNNN |
|  | RT8 | CGTCCAGGCACAATCCAGTCNNNNNNN |
| Barcode Primers | Primer 1 | GCCGGAGCTCTGCAGATATC |
|  | Primer 2 | GTATCGCTGGACACTGGACC |
|  | Primer 3 | CATCACATAGGCGTCCGCTG |
|  | Primer 4 | CGCAGGACCTCTGATACAGG |
|  | Primer 5 | CCGAGGTTCAAGCGAGGTTG |
|  | Primer 6 | GGTGGGCGTGTGAAATCGAC |
|  | Primer 7 | CGACCCTCTTATCGTGACGG |
|  | Primer 8 | CGTCCAGGCACAATCCAGTC |
