# Supplementary material for: Surveillance of tick-borne viruses in the border regions of the Tumen River Basin: Co-circulation in ticks and livestock
Source: PLoS Negl Trop Dis. 2025 Sep 4;19(9):e0013500. doi: 10.1371/journal.pntd.0013500 (PMC12419658; doi:10.1371/journal.pntd.0013500)
Supplement: S2 Table — (DOCX) [file pntd.0013500.s002.docx]

**S2 Table. Primers and probes used in the present study**

| Virus | Type | Genome locus | Primer/probe | Sequence (5′→3′) | Amplicon size (bp) | Reference |
| --- | --- | --- | --- | --- | --- | --- |
| DBTV | qRT-PCR | segment S | F | TGCTCCTCTCCGCACACCT | 76 | [1] |
|  |  |  | R | TGGCAAGTAGAGGAAACTGGTGA |  |  |
|  |  |  | Probe | FAM-TCCCTCCAGCCATCACCACCTCC-BHQ1 |  |  |
|  | RT-PCR | segment S | F | CTGGACCAAGGATGTGGCAT | 520 | This study |
|  |  |  | R | CAGCTTCTTGAGGCTCTGCT |  |  |
| SGLV | qRT-PCR | segment S | F | ATGGCACCTGTGTATGAG | 122 |  |
|  |  |  | R | AGGCTTTCGTACTCCTTG |  |  |
|  |  |  | Probe | FAM-ATCGTCAGGAGAAGCTTCG-BHQ1 |  |  |
|  | RT-PCR | segment S | F | CCCTCTGCACAGGAACAAA | 500 |  |
|  |  |  | R | GACCCTGTAGATCTTGACAAC |  |  |
| YGTV | RT-PCR | segment  1 | F | GGCTGGACTGGCTATGGT | 683 | This study |
|  |  |  | R | GTGCCTTGCGGGTGTAA |  |  |
|  |  | Segment  2 | F | ACTACTGGTTGCCGTCCTCG | 305 | [2] |
|  |  |  | R | GTCGCTGCAGTCAAATATCT |  |  |
|  |  | Segment  3 | F | CAACAGCAGTTCCCTTCGT | 1130 | This study |
|  |  |  | R | TGCCCTCAGTATCGTCTCAT |  |  |
|  |  | Segment  4 | F | ACAGGAGGCAAGACATACGC | 379 |  |
|  |  |  | R | GGTGTTATCTGGGTCCATTGTG |  |  |

[1] Shao L, Pang Z, Fu H, Chang R, Lin Z, Lv A, et al. Identification of recently identified tick-borne viruses (Dabieshan tick virus and SFTSV) by metagenomic analysis in ticks from Shandong Province, China. J Infect 2020;81(6):973-8. doi:10.1016/j.jinf.2020.10.022

[2] Kholodilov IS, Belova OA, Ivannikova AY, et al. Distribution and Characterisation of Tick-Borne Flavi-, Flavi-like, and Phenuiviruses in the Chelyabinsk Region of Russia.Viruses 2022;14(12):2699. Published 2022 Dec 1. doi:10.3390/v14122699
