# Supplementary material for: Surveillance of tick-borne viruses in the border regions of the Tumen River Basin: Co-circulation in ticks and livestock
Source: PLoS Negl Trop Dis. 2025 Sep 4;19(9):e0013500. doi: 10.1371/journal.pntd.0013500 (PMC12419658; doi:10.1371/journal.pntd.0013500)
Supplement: S3 Table — (DOCX) [file pntd.0013500.s003.docx]

**S3 Table. Tick species for DBTV, SGLV, and YGTV collected in Tumen River basin area, China**

| Collection site | No. of individuals/pools | *Haemaphysalis longicornis* | *Haemaphysalis concinna* | *Haemaphysalis japonica* | *Ixodes persulcatus* | *Dermacentor silvarum* |
| --- | --- | --- | --- | --- | --- | --- |
|  |  |  |  |  |  |  |
| Helong | 416/56 | − | 358/39 | 12/6 | 46/11 | − |
| Hunchun | 676/107 | 493/59 | 27/11 | 37/11 | 85/15 | 34/11 |
| Antu | 620/75 | − | 156/17 | 62/10 | 28/9 | 374/39 |
| Longjing | 292/40 | − | 234/27 | 40/7 | 2/1 | 16/5 |
| Total | 2004/278 | 493/59 | 775/94 | 151/34 | 161/36 | 424/55 |
