# Supplementary material for: Surveillance of tick-borne viruses in the border regions of the Tumen River Basin: Co-circulation in ticks and livestock
Source: PLoS Negl Trop Dis. 2025 Sep 4;19(9):e0013500. doi: 10.1371/journal.pntd.0013500 (PMC12419658; doi:10.1371/journal.pntd.0013500)
Supplement: S4 Table — (DOCX) [file pntd.0013500.s004.docx]

**S4 Table. Positive rates of tick samples for DBTV、SGLV and YGTV collected in Tumen River basin area, China**

| Collection site | Dabieshan tick virus (DBTV) | | | Songling virus (SGLV) | | | Yanggou tick virus (YGTV) | |
| --- | --- | --- | --- | --- | --- | --- | --- | --- |
|  | qRT-PCR | RT-PCR | Minimum infection rate (MIR) | qRT-PCR | RT-PCR | Minimum infection rate (MIR) | RT-PCR | Minimum infection rate (MIR) |
| Helong | 0 | 0 | 0 | 7 | 5 | 1.68% | 0 | 0 |
| Hunchun | 32 | 26 | 4.73% | 5 | 3 | 0.74% | 0 | 0 |
| Antu | 0 | 0 | 0 | 10 | 7 | 1.61% | 6 | 0.97% |
| Longjing | 0 | 0 | 0 | 14 | 10 | 4.79% | 0 | 0 |
| Total | 32 | 26 | 1.59% | 36 | 25 | 1.80% | 6 | 0.30% |
