# Supplementary material for: Surveillance of tick-borne viruses in the border regions of the Tumen River Basin: Co-circulation in ticks and livestock
Source: PLoS Negl Trop Dis. 2025 Sep 4;19(9):e0013500. doi: 10.1371/journal.pntd.0013500 (PMC12419658; doi:10.1371/journal.pntd.0013500)
Supplement: S5 Table — (DOCX) [file pntd.0013500.s005.docx]

**S5 Table. Pairwise comparison (%) of nucleotide identity for the S segment of Dabieshan tick virus in the study**

| Virus strain | 1 | 2 | 3 | 4 | 5 | 6 | 7 |
| --- | --- | --- | --- | --- | --- | --- | --- |
| 1.PV034576 Dabieshan tick virus/ JLYB-2024-2/ China | 100.0 |  |  |  |  |  |  |
| 2.LC753190 Dabieshan tick virus/ N11-12/ Japan | 99.1 | 100.0 |  |  |  |  |  |
| 3.KM114248 Uukuniemi virus/ Potepli 63/ Czech Republic | 41.7 | 41.7 | 100.0 |  |  |  |  |
| 4.NC055428 Yongjia Tick Virus 1/ YJ1-1/ China | 59.0 | 58.9 | 41.6 | 100.0 |  |  |  |
| 5.NC040492 Kaisodi virus/ G14132/ G14132/ India:Karnataka | 40.9 | 40.7 | 41.5 | 40.1 | 100.0 |  |  |
| 6.NC055334 Grand Arbaud virus/ Argas 27 /France | 39.7 | 39.6 | 63.2 | 38.7 | 41.0 | 100.0 |  |
| 7.NC055351 Murre virus/ Murre H/ USA | 40.4 | 40.3 | 59.2 | 40.9 | 41.5 | 55.8 | 100.0 |
