# Supplementary material for: Surveillance of tick-borne viruses in the border regions of the Tumen River Basin: Co-circulation in ticks and livestock
Source: PLoS Negl Trop Dis. 2025 Sep 4;19(9):e0013500. doi: 10.1371/journal.pntd.0013500 (PMC12419658; doi:10.1371/journal.pntd.0013500)
Supplement: S6 Table — (DOCX) [file pntd.0013500.s006.docx]

**S6 Table. Pairwise comparison (%) of nucleotide identity for the L segment of Dabieshan tick virus in the study**

| Virus strain | 1 | 2 | 3 | 4 | 5 | 6 | 7 |
| --- | --- | --- | --- | --- | --- | --- | --- |
| 1.PV034575 Dabieshan tick virus/ JLYB-2024-1/ China | 100.0 |  |  |  |  |  |  |
| 2.LC753182 Dabieshan tick virus/ N11-12/ Japan | 98.0 | 100.0 |  |  |  |  |  |
| 3.KM114246 Uukuniemi virus/ Potepli 63/ Czech Republic | 48.9 | 48.7 | 100.0 |  |  |  |  |
| 4.NC055427 Yongjia tick virus 1/ YJ1-1/ China | 65.4 | 65.8 | 49.0 | 100.0 |  |  |  |
| 5.NC040494 Kaisodi virus/ G14132/ India:Karnataka | 47.2 | 47.3 | 54.5 | 47.4 | 100.0 |  |  |
| 6.NC055333 Grand arbaud virus/ Argas 27/ France | 47.6 | 47.9 | 70.8 | 47.6 | 53.7 | 100.0 |  |
| 7.NC055353 Murre virus/ Murre H/ USA | 47.6 | 47.5 | 70.8 | 47.5 | 54.2 | 69.9 | 100.0 |
