# Supplementary material for: Surveillance of tick-borne viruses in the border regions of the Tumen River Basin: Co-circulation in ticks and livestock
Source: PLoS Negl Trop Dis. 2025 Sep 4;19(9):e0013500. doi: 10.1371/journal.pntd.0013500 (PMC12419658; doi:10.1371/journal.pntd.0013500)
Supplement: S7 Table — (DOCX) [file pntd.0013500.s007.docx]

**S7 Table. Pairwise comparison (%) of nucleotide identity for the S segment of Songling tick virus in the study**

| Virus strain | 1 | 2 | 3 | 4 | 5 | 6 | 7 | 8 |
| --- | --- | --- | --- | --- | --- | --- | --- | --- |
| 1.PV034577 Songling virus/ JLYB-2024-3/ China | 100.0 |  |  |  |  |  |  |  |
| 2.NC079002 Songling virus/ HLJ1202/ China: Heilongjiang, Lanxi | 97.5 | 100.0 |  |  |  |  |  |  |
| 3.MT328780 Songling virus/ YC585/ China: Heilongjiang, Yichun | 98.4 | 98.8 | 100.0 |  |  |  |  |  |
| 4.ON408081 Songling virus/ NE-TH2/ China: Tahe, Heilongjiang | 97.3 | 96.9 | 97.9 | 100.0 |  |  |  |  |
| 5.NC043437 Burana virus/ 760/ Kyrgyzstan | 58.5 | 58.3 | 58.8 | 58.7 | 100.0 |  |  |  |
| 6.KM817745 Wenzhou tick virus/ TS1-2/ China | 55.8 | 55.3 | 55.9 | 55.9 | 60.5 | 100.0 |  |  |
| 7.PP945071 Orthonairovirus huangpiense/ China-NX155/ China:Ningxia | 51.3 | 50.7 | 51.2 | 51.0 | 53.4 | 50.9 | 100.0 |  |
| 8.PP260020 Tacheng tick virus/ b81/ Poland | 53.2 | 53.2 | 53.4 | 53.0 | 52.3 | 53.7 | 59.8 | 100 |
