# Supplementary material for: Surveillance of tick-borne viruses in the border regions of the Tumen River Basin: Co-circulation in ticks and livestock
Source: PLoS Negl Trop Dis. 2025 Sep 4;19(9):e0013500. doi: 10.1371/journal.pntd.0013500 (PMC12419658; doi:10.1371/journal.pntd.0013500)
Supplement: S8 Table — (DOCX) [file pntd.0013500.s008.docx]

**S8 Table. Pairwise comparison (%) of nucleotide identity for the M segment of Songling tick virus in the study**

| Virus strain | 1 | 2 | 3 | 4 | 5 | 6 | 7 | 8 |
| --- | --- | --- | --- | --- | --- | --- | --- | --- |
| 1.PV034579 Songling virus/ JLYB-2024-5/ China | 100.0 |  |  |  |  |  |  |  |
| 2.NC079000 Songling virus/ HLJ1202/ China: Heilongjiang, Lanxi | 92.6 | 100.0 |  |  |  |  |  |  |
| 3.MT328778 Songling virus/ YC585/ China: Heilongjiang, Yichun | 92.8 | 99.0 | 100.0 |  |  |  |  |  |
| 4.ON408080 Songling virus/ NE-TH2/ China: Tahe, Heilongjiang | 94.9 | 91.7 | 91.9 | 100.0 |  |  |  |  |
| 5.NC043438 Burana virus/ 760/ Kyrgyzstan | 60.5 | 60.3 | 60.4 | 60.5 | 100.0 |  |  |  |
| 6.KM817718 Wenzhou tick virus/ TS1-2/ China | 60.9 | 61.0 | 61.2 | 60.9 | 57.9 | 100.0 |  |  |
| 7.PP945070 Orthonairovirus huangpiense/ China-NX155/ China:Ningxia | 52.7 | 52.2 | 52.6 | 52.4 | 53.7 | 50.9 | 100.0 |  |
| 8.PP260018 Tacheng tick virus/ b81/ Poland | 60.9 | 60.3 | 60.8 | 61.2 | 61.9 | 60.7 | 57.7 | 100 |
