# Supplementary material for: Surveillance of tick-borne viruses in the border regions of the Tumen River Basin: Co-circulation in ticks and livestock
Source: PLoS Negl Trop Dis. 2025 Sep 4;19(9):e0013500. doi: 10.1371/journal.pntd.0013500 (PMC12419658; doi:10.1371/journal.pntd.0013500)
Supplement: S9 Table — (DOCX) [file pntd.0013500.s009.docx]

**S9 Table. Pairwise comparison (%) of nucleotide identity for the L segment of Songling tick virus in the study**

| Virus strain | 1 | 2 | 3 | 4 | 5 | 6 | 7 | 8 |
| --- | --- | --- | --- | --- | --- | --- | --- | --- |
| 1.PV034578 Songling virus/ JLYB-2024-4/ China | 100.0 |  |  |  |  |  |  |  |
| 2.NC079001 Songling virus/ HLJ1202/ China: Heilongjiang, Lanxi | 96.3 | 100.0 |  |  |  |  |  |  |
| 3.MT328779 Songling virus/ YC585/ China: Heilongjiang, Yichun | 96.8 | 98.5 | 100.0 |  |  |  |  |  |
| 4.ON408079 Songling virus/ NE-TH2/ China: Tahe, Heilongjiang | 97.0 | 96.4 | 96.9 | 100.0 |  |  |  |  |
| 5.NC043439 Burana virus/ 760/ Kyrgyzstan | 63.4 | 63.3 | 63.5 | 63.7 | 100.0 |  |  |  |
| 6.KM817685 Wenzhou tick virus/ TS1-2/ China | 63.4 | 63.3 | 63.5 | 63.6 | 65.8 | 100.0 |  |  |
| 7.PP945069 Orthonairovirus huangpiense/ China-NX155/ China:Ningxia | 59.8 | 59.9 | 60.1 | 59.9 | 60.5 | 59.5 | 100.0 |  |
| 8.PP260015 Tacheng tick virus/ b81/ Poland | 62.9 | 62.6 | 62.7 | 62.8 | 62.4 | 61.9 | 60.4 | 100 |
