# Supplementary material for: Surveillance of tick-borne viruses in the border regions of the Tumen River Basin: Co-circulation in ticks and livestock
Source: PLoS Negl Trop Dis. 2025 Sep 4;19(9):e0013500. doi: 10.1371/journal.pntd.0013500 (PMC12419658; doi:10.1371/journal.pntd.0013500)
Supplement: S10 Table — (DOCX) [file pntd.0013500.s010.docx]

**S10 Table. Pairwise comparison (%) of nucleotide identity for the protein S segment of Songling tick virus in the study**

| Virus strain | 1 | 2 | 3 | 4 | 5 | 6 | 7 | 8 |
| --- | --- | --- | --- | --- | --- | --- | --- | --- |
| 1.PV034568 Songling virus/ JLYB-2024-YX1/ China | 100.0 |  |  |  |  |  |  |  |
| 2.PV034569 Songling virus/ JLYB-2024-60T/ China | 98.7 | 100.0 |  |  |  |  |  |  |
| 3.PV034570 Songling virus/ JLYB-2024-67T/ China | 96.4 | 99.6 | 100.0 |  |  |  |  |  |
| 4.ON408081 Songling virus/ NE-TH2/ China: Tahe, Heilongjiang | 99.0 | 99.1 | 96.8 | 100.0 |  |  |  |  |
| 5.NC079002 Songling virus/ HLJ1202/ China: Heilongjiang, Lanxi | 98.0 | 97.8 | 95.5 | 98.2 | 100.0 |  |  |  |
| 6.MT815994 Tamdy virus/ YL16083/ China | 42.1 | 41.6 | 41.3 | 41.5 | 41.5 | 100.0 |  |  |
| 7.ON408090 Ji'an nariovirus/ NE-JA/ China: Ji'an, Jilin | 41.9 | 41.8 | 41.6 | 42.1 | 41.7 | 57.8 | 100.0 |  |
| 8.OQ207701 Antu virus/ YB tick 2021 24/ China: Yanbian, Antu | 41.7 | 41.6 | 41.4 | 41.9 | 41.5 | 57.7 | 98.8 | 100 |
