# Supplementary material for: Surveillance of tick-borne viruses in the border regions of the Tumen River Basin: Co-circulation in ticks and livestock
Source: PLoS Negl Trop Dis. 2025 Sep 4;19(9):e0013500. doi: 10.1371/journal.pntd.0013500 (PMC12419658; doi:10.1371/journal.pntd.0013500)
Supplement: S11 Table — (DOCX) [file pntd.0013500.s011.docx]

**S11 Table. Pairwise comparison (%) of nucleotide identity for the protein 1 segment of Yanggou tick virus in the study.**

| Virus strain | 1 | 2 | 3 | 4 | 5 | 6 | 7 | 8 | 9 | 10 | 11 | 12 | 13 | 14 | 15 | 16 |
| --- | --- | --- | --- | --- | --- | --- | --- | --- | --- | --- | --- | --- | --- | --- | --- | --- |
| 1.PV951568 Yanggou tick virus/ T1732/ China | 100.0 |  |  |  |  |  |  |  |  |  |  |  |  |  |  |  |
| 2.PV951567 Yanggou tick virus/ T1662/ China | 98.9 | 100.0 |  |  |  |  |  |  |  |  |  |  |  |  |  |  |
| 3.PV941017 Yanggou tick virus/ T117/ China | 98.9 | 100.0 | 100.0 |  |  |  |  |  |  |  |  |  |  |  |  |  |
| 4.PV941021 Yanggou tick virus/ N177/ China | 99.4 | 99.5 | 99.5 | 100.0 |  |  |  |  |  |  |  |  |  |  |  |  |
| 5.MT24841 Yanggou tick virus/ XJ-YGTV-1/ China | 92.2 | 92.7 | 92.7 | 92.8 | 100.0 |  |  |  |  |  |  |  |  |  |  |  |
| 6.OR148890 Yanggou tick virus/ YGTV YBQG1718A/ China: Yanbian | 97.1 | 97.9 | 97.9 | 97.7 | 94.8 | 100.0 |  |  |  |  |  |  |  |  |  |  |
| 7.MW556730 Yanggou tick virus/ Republic Altay/997/2016/ Russia: Republic Altay | 93.6 | 93.7 | 93.7 | 94.2 | 96.3 | 94.7 | 100.0 |  |  |  |  |  |  |  |  |  |
| 8.MW525322 Yanggou tick virus/ Erzin14-T20074/ Russia: Republic of Tuva | 96.8 | 97.3 | 97.3 | 97.4 | 92.1 | 96.3 | 92.8 | 100.0 |  |  |  |  |  |  |  |  |
| 9.PP125351 Yanggou tick virus/ Mongolia b77/ Mongolia | 96.3 | 96.8 | 96.8 | 96.9 | 92.5 | 96.2 | 93.0 | 97.1 | 100.0 |  |  |  |  |  |  |  |
| 10.MH688536 Yanggou tick virus/ 17-L1/ China | 92.7 | 93.1 | 93.1 | 93.3 | 99.5 | 95.3 | 96.8 | 92.5 | 93.0 | 100.0 |  |  |  |  |  |  |
| 11.MH688529 Yanggou tick virus/ YG / China | 92.2 | 92.7 | 92.7 | 92.8 | 100.0 | 94.8 | 96.3 | 92.1 | 92.5 | 99.5 | 100.0 |  |  |  |  |  |
| 12.NC024113 Jingmen tick virus/ SY84/ China | 72.7 | 72.8 | 72.8 | 73.3 | 71.9 | 72.5 | 72.4 | 72.2 | 72.2 | 72.1 | 71.9 | 100.0 |  |  |  |  |
| 13.MK721860 Heilongjiang tick virus/ HLJ41/ China | 71.9 | 72.1 | 72.1 | 72.5 | 70.4 | 71.8 | 70.8 | 71.5 | 70.5 | 70.5 | 70.4 | 94.7 | 100.0 |  |  |  |
| 14.MK721856 Guangxi tick virus/ GX46/ China | 71.9 | 72.1 | 72.1 | 72.5 | 70.4 | 71.8 | 70.8 | 71.5 | 70.5 | 70.5 | 70.4 | 94.7 | 100.0 | 100.0 |  |  |
| 15.OQ158902 SCWL tick virus/ PC-18 / China: Sichuan Wolong | 72.1 | 72.5 | 72.5 | 72.7 | 70.5 | 72.2 | 71.5 | 71.6 | 70.7 | 70.7 | 70.5 | 93.6 | 95.0 | 95.0 | 100.0 |  |
| 16.OQ320759 Sichuan tick virus/ PC-16/ China: Sichuan Wolong | 72.1 | 72.5 | 72.5 | 72.7 | 70.5 | 72.2 | 71.5 | 71.6 | 70.7 | 70.7 | 70.5 | 93.6 | 95.0 | 95.0 | 100.0 | 100.0 |
