# Supplementary material for: Surveillance of tick-borne viruses in the border regions of the Tumen River Basin: Co-circulation in ticks and livestock
Source: PLoS Negl Trop Dis. 2025 Sep 4;19(9):e0013500. doi: 10.1371/journal.pntd.0013500 (PMC12419658; doi:10.1371/journal.pntd.0013500)
Supplement: S12 Table — (DOCX) [file pntd.0013500.s012.docx]

**S12 Table. Pairwise comparison (%) of nucleotide identity for the protein 2 segment of Yanggou tick virus in the study.**

| Virus strain | 1 | 2 | 3 | 4 | 5 | 6 | 7 | 8 | 9 | 10 | 11 | 12 | 13 | 14 | 15 | 16 | 17 | 18 |
| --- | --- | --- | --- | --- | --- | --- | --- | --- | --- | --- | --- | --- | --- | --- | --- | --- | --- | --- |
| 1.PV034571 Yanggou tick virus/ JLYB-2024-47N/ China | 100.0 |  |  |  |  |  |  |  |  |  |  |  |  |  |  |  |  |  |
| 2.PV034572 Yanggou tick virus/ JLYB-2024-153-3T/ China | 95.7 | 100.0 |  |  |  |  |  |  |  |  |  |  |  |  |  |  |  |  |
| 3.PV034573 Yanggou tick virus/ JLYB-2024-153-6T/ China | 95.4 | 97.8 | 100.0 |  |  |  |  |  |  |  |  |  |  |  |  |  |  |  |
| 4.PV034574 Yanggou tick virus/ JLYB-2024-153-1T/ China | 96.3 | 98.8 | 99.1 | 100.0 |  |  |  |  |  |  |  |  |  |  |  |  |  |  |
| 5.OR148891 Yanggou tick virus/ YGTV YBQG1718A/ China: Yanbian | 96.9 | 98.2 | 97.8 | 98.8 | 100.0 |  |  |  |  |  |  |  |  |  |  |  |  |  |
| 6.MW525323 Yanggou tick virus/ Erzin14-T20074/ Russia: Republic of Tuva | 95.7 | 97.5 | 96.6 | 97.5 | 98.8 | 100.0 |  |  |  |  |  |  |  |  |  |  |  |  |
| 7.OP125783 Yanggou tick virus/ Bredy14-T19741/ Russia: Chelyabinsk region | 94.8 | 96.0 | 95.1 | 96.0 | 97.2 | 97.2 | 100.0 |  |  |  |  |  |  |  |  |  |  |  |
| 8.OP125799 Yanggou tick virus/ Zaozernyy 15-T22264/ Russia: Chelyabinsk region | 94.8 | 96.0 | 95.1 | 96.0 | 97.2 | 97.2 | 100.0 | 100.0 |  |  |  |  |  |  |  |  |  |  |
| 9.OP125794 Yanggou tick virus/ Kartaly14-T19551/ Russia: Chelyabinsk region | 94.8 | 96.0 | 95.1 | 96.0 | 97.2 | 97.2 | 100.0 | 100.0 | 100.0 |  |  |  |  |  |  |  |  |  |
| 10.OP125784 Yanggou tick virus/ Bredy14-T19767/ Russia: Chelyabinsk region | 94.5 | 95.7 | 94.8 | 95.7 | 96.9 | 96.9 | 99.1 | 99.1 | 99.1 | 100.0 |  |  |  |  |  |  |  |  |
| 11.MH688537 Yanggou tick virus/ 17-L1/ China | 91.4 | 92.1 | 91.4 | 92.1 | 92.8 | 92.8 | 94.4 | 94.4 | 94.4 | 93.8 | 100.0 |  |  |  |  |  |  |  |
| 12.MT248419 Yanggou tick virus/ XJ-YGTV-1/ China | 91.1 | 91.8 | 91.1 | 91.8 | 92.4 | 92.4 | 94.1 | 94.1 | 94.1 | 93.4 | 99.0 | 100.0 |  |  |  |  |  |  |
| 13.MH688530 Yanggou tick virus/ YG/ China | 91.1 | 91.8 | 91.1 | 91.8 | 92.4 | 92.4 | 94.1 | 94.1 | 94.1 | 93.4 | 99.0 | 100.0 | 100.0 |  |  |  |  |  |
| 14.OQ320760 Sichuan tick virus/ PC-16/ China: Sichuan Wolong | 50.6 | 49.7 | 49.7 | 49.7 | 50.0 | 50.3 | 51.3 | 51.3 | 51.3 | 50.9 | 53.9 | 53.9 | 53.9 | 100.0 |  |  |  |  |
| 15.OQ158903 SCWL tick virus/ PC-18/ China: Sichuan Wolong | 50.3 | 49.4 | 49.4 | 49.4 | 49.7 | 50.0 | 50.9 | 50.9 | 50.9 | 50.6 | 53.6 | 53.6 | 53.6 | 99.9 | 100.0 |  |  |  |
| 16.NC024112 Jingmen tick virus/ SY84/ China | 51.6 | 50.6 | 50.6 | 50.6 | 50.9 | 51.3 | 52.2 | 52.2 | 52.2 | 51.9 | 55.3 | 54.9 | 54.9 | 92.6 | 92.9 | 100.0 |  |  |
| 17.MK721857 Guangxi tick virus/ GX46/ China | 50.9 | 50.0 | 50.0 | 50.0 | 50.3 | 50.6 | 51.6 | 51.6 | 51.6 | 51.3 | 55.3 | 54.9 | 54.9 | 93.1 | 93.4 | 94.2 | 100.0 |  |
| 18.MK721861 Heilongjiang tick virus/ HLJ41/ China | 50.9 | 50.0 | 50.0 | 50.0 | 50.3 | 50.6 | 51.6 | 51.6 | 51.6 | 51.3 | 55.3 | 54.9 | 54.9 | 93.1 | 93.4 | 94.2 | 100.0 | 100.0 |
