# Supplementary material for: Surveillance of tick-borne viruses in the border regions of the Tumen River Basin: Co-circulation in ticks and livestock
Source: PLoS Negl Trop Dis. 2025 Sep 4;19(9):e0013500. doi: 10.1371/journal.pntd.0013500 (PMC12419658; doi:10.1371/journal.pntd.0013500)
Supplement: S13 Table — (DOCX) [file pntd.0013500.s013.docx]

**S13 Table. Pairwise comparison (%) of nucleotide identity for the protein 3 segment of Yanggou tick virus in the study.**

| Virus strain | 1 | 2 | 3 | 4 | 5 | 6 | 7 | 8 | 9 | 10 | 11 | 12 | 13 | 14 | 15 | 16 |
| --- | --- | --- | --- | --- | --- | --- | --- | --- | --- | --- | --- | --- | --- | --- | --- | --- |
| 1.PV921634 Yanggou tick virus/ N3151/ China | 100.0 |  |  |  |  |  |  |  |  |  |  |  |  |  |  |  |
| 2.PV921633 Yanggou tick virus/ T31/ China | 99.8 | 100.0 |  |  |  |  |  |  |  |  |  |  |  |  |  |  |
| 3.PV921635 Yanggou tick virus/ T312/ China | 99.8 | 100.0 | 100.0 |  |  |  |  |  |  |  |  |  |  |  |  |  |
| 4.PV921636 Yanggou tick virus/ T3111/ China | 99.8 | 100.0 | 100.0 | 100.0 |  |  |  |  |  |  |  |  |  |  |  |  |
| 5.MH688538 Yanggou tick virus/ 17-L1/ China | 97.1 | 97.3 | 97.3 | 97.3 | 100.0 |  |  |  |  |  |  |  |  |  |  |  |
| 6.MH688534 Yanggou tick virus/ 16-T2/ China | 97.1 | 97.3 | 97.3 | 97.3 | 100.0 | 100.0 |  |  |  |  |  |  |  |  |  |  |
| 7.MH688531 Yanggou tick virus/ YG/ China | 97.0 | 97.2 | 97.2 | 97.2 | 99.9 | 99.9 | 100.0 |  |  |  |  |  |  |  |  |  |
| 8.MT248420 Yanggou tick virus/ XJ-YGTV-1/ China | 96.5 | 96.7 | 96.6 | 96.7 | 99.4 | 99.4 | 99.4 | 100.0 |  |  |  |  |  |  |  |  |
| 9.OR148892 Yanggou tick virus/ YGTV YBQG1718A/ China: Yanbian | 99.3 | 99.5 | 99.5 | 99.5 | 97.0 | 97.0 | 96.9 | 96.5 | 100.0 |  |  |  |  |  |  |  |
| 10.MW556732 Yanggou tick virus/ Republic Altay/997/2016/ Russia: Republic Altay | 93.4 | 93.6 | 93.5 | 93.6 | 93.1 | 93.1 | 93.3 | 92.8 | 93.1 | 100.0 |  |  |  |  |  |  |
| 11.MW525324 Yanggou tick virus/ Erzin14-T20074/ Russia: Republic of Tuva | 95.2 | 95.4 | 95.4 | 95.4 | 95.6 | 95.6 | 95.7 | 95.3 | 95.0 | 93.6 | 100.0 |  |  |  |  |  |
| 12. NC024114 Jingmen tick virus/ SY84/ China | 66.7 | 67.9 | 67.7 | 66.9 | 70.1 | 69.9 | 70.1 | 69.8 | 70.2 | 70.3 | 69.0 | 100.0 |  |  |  |  |
| 13.MK721858 Guangxi tick virus/ GX46/ China | 65.6 | 66.9 | 66.6 | 65.8 | 69.6 | 69.4 | 69.6 | 69.6 | 69.1 | 69.1 | 68.2 | 94.4 | 100.0 |  |  |  |
| 14.MK721862 Heilongjiang tick virus/ HLJ41/ China | 65.6 | 66.9 | 66.6 | 65.8 | 69.6 | 69.4 | 69.6 | 69.6 | 69.1 | 69.1 | 68.2 | 94.4 | 100.0 | 100.0 |  |  |
| 15.OQ158904 SCWL tick virus/ PC-18/ China: Sichuan Wolong | 66.4 | 67.7 | 67.6 | 66.6 | 69.8 | 69.7 | 69.8 | 69.5 | 69.5 | 70.2 | 68.6 | 94.1 | 94.1 | 94.1 | 100.0 |  |
| 16.OQ320761 Sichuan tick virus/ PC-16/ China: Sichuan Wolong | 66.4 | 67.7 | 67.6 | 66.6 | 69.8 | 69.7 | 69.8 | 69.5 | 69.5 | 70.2 | 68.6 | 94.0 | 94.1 | 94.0 | 99.9 | 100.0 |
