# Supplementary material for: Surveillance of tick-borne viruses in the border regions of the Tumen River Basin: Co-circulation in ticks and livestock
Source: PLoS Negl Trop Dis. 2025 Sep 4;19(9):e0013500. doi: 10.1371/journal.pntd.0013500 (PMC12419658; doi:10.1371/journal.pntd.0013500)
Supplement: S14 Table — (DOCX) [file pntd.0013500.s014.docx]

**S14 Table. Pairwise comparison (%) of nucleotide identity for the protein 4 segment of Yanggou tick virus in the study.**

| Virus strain | 1 | 2 | 3 | 4 | 5 | 6 | 7 | 8 | 9 | 10 | 11 | 12 | 13 | 14 | 15 | 16 |
| --- | --- | --- | --- | --- | --- | --- | --- | --- | --- | --- | --- | --- | --- | --- | --- | --- |
| 1.PV941020 Yanggou tick virus/ 387N/ China | 100.0 |  |  |  |  |  |  |  |  |  |  |  |  |  |  |  |
| 2.PV941015 Yanggou tick virus/ 337T/ China | 100.0 | 100.0 |  |  |  |  |  |  |  |  |  |  |  |  |  |  |
| 3.PV941014 Yanggou tick virus/ 327T/ China | 99.7 | 99.7 | 100.0 |  |  |  |  |  |  |  |  |  |  |  |  |  |
| 4.PV941013 Yanggou tick virus/ 318T/ China | 100.0 | 100.0 | 100.0 | 100.0 |  |  |  |  |  |  |  |  |  |  |  |  |
| 5.OR148893 Yanggou tick virus/ YGTV YBQG1718A/ China: Yanbian | 99.7 | 99.7 | 99.7 | 99.7 | 100.0 |  |  |  |  |  |  |  |  |  |  |  |
| 6.MW556733 Yanggou tick virus/ Republic Altay/997/2016/ Russia: Republic Altay | 94.3 | 94.3 | 94.3 | 94.3 | 94.6 | 100.0 |  |  |  |  |  |  |  |  |  |  |
| 7.PP125353 Yanggou tick virus/ Mongolia b77/ Mongolia | 96.3 | 96.3 | 96.3 | 96.3 | 96.0 | 93.5 | 100.0 |  |  |  |  |  |  |  |  |  |
| 8.MH688532 Yanggou tick virus/ YG/ China | 94.6 | 94.6 | 94.6 | 94.6 | 94.9 | 96.0 | 94.3 | 100.0 |  |  |  |  |  |  |  |  |
| 9.MT248421 Yanggou tick virus/ XJ-YGTV-1/ China | 94.6 | 94.6 | 94.6 | 94.6 | 94.9 | 96.0 | 94.3 | 100.0 | 100.0 |  |  |  |  |  |  |  |
| 10.MH688539 Yanggou tick virus/ 17-L1/ China | 94.6 | 94.6 | 94.6 | 94.6 | 94.9 | 96.0 | 94.3 | 98.9 | 98.9 | 100.0 |  |  |  |  |  |  |
| 11.MW525325 Yanggou tick virus/ Erzin14-T20074/ Russia: Republic of Tuva | 95.7 | 95.7 | 95.7 | 95.7 | 95.4 | 93.7 | 98.3 | 94.8 | 94.8 | 94.8 | 100.0 |  |  |  |  |  |
| 12.MK721859 Guangxi tick virus/ GX46/ China | 67.2 | 67.1 | 66.3 | 67.0 | 67.3 | 66.8 | 67.3 | 67.6 | 67.6 | 67.6 | 68.2 | 100.0 |  |  |  |  |
| 13.MK721863 Heilongjiang tick virus/ HLJ41/ China | 67.2 | 67.1 | 66.3 | 67.0 | 67.3 | 66.8 | 67.3 | 67.6 | 67.6 | 67.6 | 68.2 | 100.0 | 100.0 |  |  |  |
| 14.OQ320762 Sichuan tick virus/ PC-16/ China: Sichuan Wolong | 66.7 | 66.6 | 66.0 | 66.5 | 66.8 | 65.3 | 66.2 | 66.8 | 66.8 | 66.2 | 66.5 | 94.3 | 94.3 | 100.0 |  |  |
| 15.OQ158905 SCWL tick virus/ China: Sichuan Wolong | 66.7 | 66.6 | 66.0 | 66.5 | 66.8 | 65.3 | 66.2 | 66.8 | 66.8 | 66.2 | 66.5 | 94.1 | 93.9 | 99.8 | 100.0 |  |
| 16.NC024111 Jingmen tick virus/ SY84/ China | 66.1 | 66.0 | 65.2 | 65.9 | 66.2 | 64.5 | 65.1 | 65.3 | 65.3 | 65.3 | 65.9 | 93.0 | 93.0 | 93.1 | 92.6 | 100.0 |
